# Impact of Query Language on the Structure and Guideline Alignment of AI-Generated Rehabilitation Programs in Chronic Kidney Disease

**DOI:** 10.3390/jcm15114218

**Published:** 2026-05-29

**Authors:** Volodymyr Bezruk, Dmytro Ivanov, Maria Ivanchuk, Igor Shkrobanets, Olena Makarova, Larysa Rynzhuk, Tetiana Bulyk, Oleg Maksymiv, Mariia Ivanova

**Affiliations:** 1 Department of Pediatrics, Neonatology and Perinatal Medicine, Bukovinian State Medical University, 58002 Chernivtsi, Ukraine; bezruk@bsmu.edu.ua; 2 Department of Nephrology and ET, Bogomolets National Medical University, 01601 Kyiv, Ukraine; 3 Department of Medical and Biological Physics and Medical Informatics, Bukovinian State Medical University, 58002 Chernivtsi, Ukraine; ivanchuk.m@bsmu.edu.ua; 4 Marzieiev Institute for Public Health of the National Academy of Medical Sciences of Ukraine, 02094 Kyiv, Ukraine; 5 Department of Nursing Care and Higher Nursing Education, Bukovinian State Medical University, 58002 Chernivtsi, Ukraine; 6 Department of Obstetrics and Gynecology, Bukovinian State Medical University, 58002 Chernivtsi, Ukraine; 7 Department of Prosthodontics, Bukovinian State Medical University, 58002 Chernivtsi, Ukraine; 8 IRCCS Ospedale San Raffaele, 20132 Milan, Italy; mesangium88@gmail.com

**Keywords:** chronic kidney disease, rehabilitation, artificial intelligence, language bias, exercise therapy, multilingual AI, prompt engineering, large language models

## Abstract

**Background:** Artificial intelligence (AI) is increasingly used in nephrology, including rehabilitation planning for patients with chronic kidney disease (CKD). However, most AI systems are predominantly trained on English-language data, which may influence the quality and clinical relevance of the generated recommendations. **Objective:** To evaluate the impact of query language on the quality and clinical applicability of AI-generated exercise programs for CKD patients undergoing renal replacement therapy. **Methods:** We conducted a structured qualitative comparison using predefined evaluation criteria based on KDIGO and ERA rehabilitation guidelines. Outputs were assessed for structure, clinical detail, safety framing, and adaptability. Identical prompts were formulated in Ukrainian and English. Generated exercise programs were assessed for alignment with international guidelines (KDIGO, ERA), level of clinical detail, progression, safety considerations, and adaptability. **Results:** All AI systems produced safe exercise programs incorporating aerobic, resistance, flexibility, and relaxation components. However, significant differences were observed depending on the query language. Ukrainian-language outputs were simpler and focused on general well-being, with limited progression and monitoring. In contrast, English-language outputs demonstrated greater clinical depth, including structured progression, intradialytic adaptations, and the use of validated monitoring tools (e.g., Borg RPE scale). Copilot provided the highest clinical precision, ChatGPT delivered structured programs, and Gemini emphasized safety and motivation. English-language prompts produced more detailed and guideline-aligned outputs, whereas Ukrainian-language prompts generated simpler, wellness-oriented recommendations. **Conclusions:** Query language influences the structure and clinical completeness of AI-generated rehabilitation programs. English-language prompts currently yield more detailed and guideline-aligned outputs. Further multilingual model development is needed. English-language queries currently yield more clinically robust outputs. Development of multilingual AI systems and standardized prompt frameworks is essential to ensure equitable access to AI-assisted healthcare.

## 1. Introduction

Artificial intelligence (AI) is rapidly transforming clinical medicine by supporting diagnostics, risk stratification, decision support, and personalized care pathways [1,2,3,4,5]. In nephrology, AI applications are expanding across imaging, prediction modeling, clinical workflow optimization, and patient-oriented digital tools, including rehabilitation planning [4,5,6,7,8]. This trend is particularly relevant for chronic kidney disease (CKD), which remains a major and growing contributor to global morbidity and mortality [9].

Exercise-based rehabilitation is increasingly recognized as an essential component of CKD management. In patients receiving hemodialysis, appropriately prescribed physical activity may improve functional capacity, reduce symptom burden, enhance cardiovascular health, and improve quality of life [10,11,12,13,14]. Recent systematic reviews and meta-analyses further confirm the clinical benefits of structured exercise interventions in CKD populations [12,15]. Despite this evidence, implementation remains inconsistent across nephrology settings, and exercise support continues to be underused in routine care [13,16,17,18]. Digital tools, including AI systems, may therefore offer scalable support for generating structured and individualized rehabilitation recommendations.

At the same time, the clinical value of generative AI depends not only on model capability but also on language, training data composition, and prompt design. Most large language models are trained predominantly on English-language corpora, raising concerns about unequal performance across languages and healthcare contexts [17,19,20,21,22]. In medicine, such linguistic asymmetry may influence completeness, safety framing, terminology, and adherence to evidence-based recommendations, potentially creating a new layer of digital inequity for non-English-speaking patients and clinicians [20,21].

Despite the rapid expansion of AI in nephrology, no studies have systematically examined how query language affects the structure and clinical completeness of AI-generated rehabilitation recommendations. This gap is particularly relevant for Ukrainian-speaking clinicians, given the limited availability of Ukrainian-language training data in current AI systems [23,24]. These issues are especially important in Eastern European countries, where clinicians increasingly use AI tools in local languages, while the strongest evidence base and model training environment remain predominantly English-language [23,25].

To address this gap, we investigated whether the language of the user query influences the clinical depth, structure, and guideline alignment of AI-generated rehabilitation programs for CKD patients. We hypothesized that English-language prompts would produce more clinically detailed and guideline-aligned outputs compared with Ukrainian-language prompts.

### Aim of the Study

The aim of this study was to systematically compare AI-generated exercise programs for patients with chronic kidney disease (CKD) receiving renal replacement therapy using Ukrainian- and English-language prompts, and to evaluate their clinical applicability, safety framing, and alignment with international rehabilitation standards.

## 2. Materials and Methods

This study was conducted within the framework of institutional research projects at Bukovinian State Medical University (State Registration No. 0122U002245, 2022–2026; No. 0125U001331, 2025–2029).

### 2.1. Study Design

We performed a cross-language comparative experimental study designed to evaluate differences in AI-generated rehabilitation recommendations depending on the language of the user query. The study consisted of three sequential components:

(1) a scientometric review to identify evidence-based rehabilitation principles in chronic kidney disease (CKD),
(2) standardized interaction with selected AI systems, and
(3) structured qualitative evaluation of generated outputs.

A methodological flow diagram is provided in the Supplementary Materials.

### 2.2. AI Systems and Model Specifications

Three widely used large language model (LLM) systems were included: Gemini (2.5 flash), ChatGPT (GPT-4.1, OpenAI), and Copilot (GPT-4-class model).

For each system, we documented:

- the model version available at the time of access,
- the date and time of interaction,
- default system settings, including temperature and generation mode.

These details are summarized in Supplementary Table S1.

All outputs were generated in a single attempt without iterative refinement. Before each query, the chat history was cleared to avoid contextual contamination. All systems were accessed in their default configurations, and no temperature or randomness adjustments were available beyond the platform defaults. Each model was queried independently under identical conditions, using the same device, browser session, and network environment to ensure comparability.

### 2.3. Prompt Design and Cross-Language Framework

To isolate the effect of query language, we used a single standardized prompt in two linguistically equivalent versions. Translation fidelity was ensured through independent forward translation and back-translation by bilingual experts.

Ukrainian prompt: «Запропонуйте безпечний комплекс фізичних вправ для пацієнтів із ХХН на гемодіалізі».

English prompt: “Please propose a safe and effective exercise program for patients with CKD on hemodialysis.”

Although the Ukrainian prompt uses the phrase “безпечний комплекс фізичних вправ,” and the English prompt specifies “safe and effective exercise program,” both were validated through forward translation and back-translation by bilingual experts. The semantic intent was confirmed to be equivalent. Nevertheless, we acknowledge that subtle lexical differences may influence model behavior, and this is addressed in the Limitations.

Each AI system was queried separately in both languages under identical conditions. We acknowledge that using a single prompt limits generalizability; this is addressed in the Limitations section.

### 2.4. Endpoints

Primary endpoint: differences in guideline alignment and clinical detail between English- and Ukrainian-language outputs.

Secondary endpoints: differences in safety framing, adaptability (intradialytic vs. home-based exercise), and structural completeness of the generated programs.

### 2.5. Evaluation Framework

A structured rubric was developed based on international nephrology and rehabilitation guidelines (KDIGO, ERA, ISN). Each AI-generated output was evaluated across the following domains:

- Guideline alignment: consistency with evidence-based CKD rehabilitation principles.
- Clinical detail: specification of frequency, duration, intensity, progression, and monitoring tools.
- Safety considerations: protection of vascular access, symptom monitoring, contraindications.
- Adaptability: inclusion of intradialytic options, home-based modifications, and patient-specific adjustments.
- Structural completeness: presence of aerobic, resistance, flexibility, balance, and relaxation components.

The full rubric is provided in Supplementary Table S2.

To enhance methodological transparency, a structured scoring matrix was applied to each domain of the rubric. Each criterion was rated on a 0–2 scale (0 = absent, 1 = partially present, 2 = fully present). Three evaluators independently scored all outputs, and discrepancies were resolved through consensus discussion. Although formal inter-rater statistics were not calculated, agreement was high across domains.

### 2.6. Evaluation Procedure

Three independent evaluators—a nephrologist, a rehabilitation specialist, and a clinical researcher—assessed all outputs. Evaluators were blinded to the identity of the AI system but not to the language of the output, which is an inherent limitation of the design. Discrepancies were resolved through consensus.

The generated outputs were evaluated independently according to predefined criteria [10,11,12,14,26,27] derived from international nephrology guidelines (KDIGO, ERA, ISN) and rehabilitation frameworks.

### 2.7. Data Analysis

Given the qualitative nature of the study, no inferential statistical testing was performed. Findings are presented descriptively using comparative tables and thematic synthesis.

### 2.8. Use of Generative AI Tools

Generative artificial intelligence tools ChatGPT (GPT-4.1, OpenAI); and Microsoft Copilot (GPT-4-class model) were used exclusively for language editing, improving clarity, and refining the structure of the manuscript. These tools were not used to generate scientific data, analyze results, or draw conclusions. All content was critically reviewed, verified, and approved by the authors.

## 3. Results

In accordance with the study objectives, three artificial intelligence (AI) systems, Gemini (2.5 flash), ChatGPT (GPT-4.1, OpenAI), and Copilot (GPT-4-class model), were evaluated for their ability to generate clinically applicable exercise programs for patients with chronic kidney disease (CKD) undergoing hemodialysis. Across all systems, the generated outputs demonstrated a consistent baseline orientation toward safety, including recommendations for prior consultation with a physician and supervision by healthcare professionals. This indicates that all evaluated models were capable of producing broadly safe, non-harmful exercise guidance.

At the same time, substantial and systematic differences were identified in the structure, clinical depth, safety framing, and adaptability of the proposed exercise programs. These differences were primarily associated with the language of the query rather than the AI system itself and were consistently observed across all three platforms. English-language prompts yielded more detailed and clinically structured outputs, whereas Ukrainian-language prompts produced simpler, less explicit, and more wellness-oriented recommendations.

**Figure 1:**
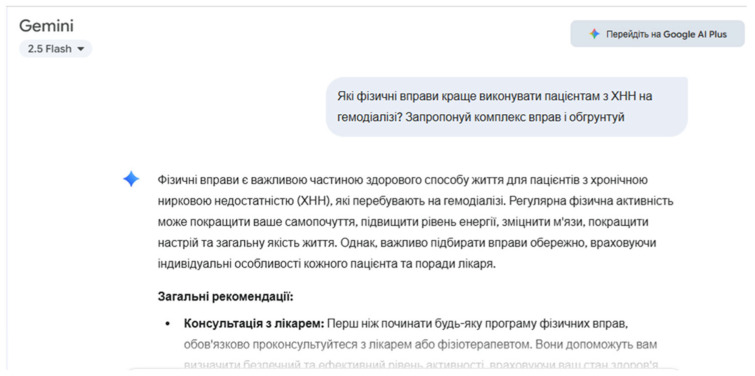
Example of an exercise program generated by the Gemini AI system in response to a Ukrainian-language prompt for patients with chronic kidney disease undergoing hemodialysis. The output demonstrates a simplified structure with general recommendations on aerobic, resistance, and flexibility exercises, without detailed progression parameters or objective intensity monitoring.

**Figure 2:**
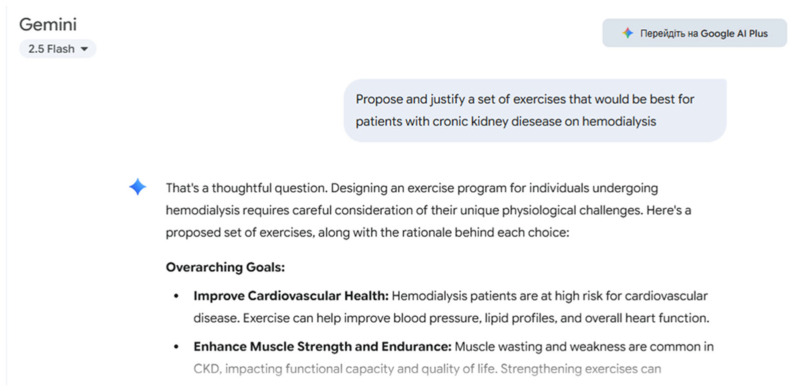
Example of an exercise program generated by the Gemini AI system in response to an English-language prompt. Compared with the Ukrainian-language version, the output demonstrates increased clinical detail, including structured progression, defined intensity levels based on the Borg scale, and expanded safety considerations.

**Figure 3:**
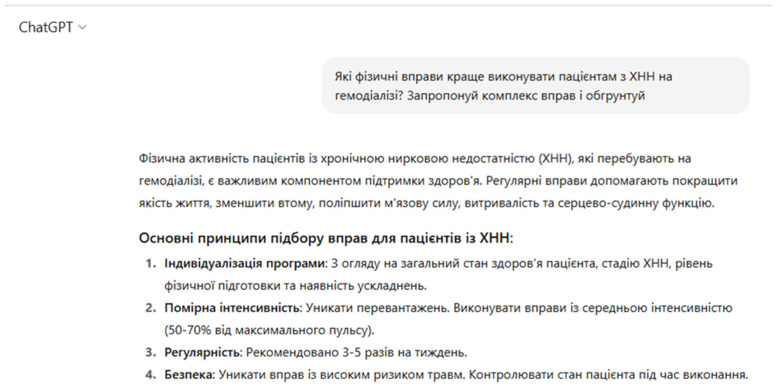
Example of an exercise program generated by ChatGPT in response to a Ukrainian-language prompt. The output presents a structured but generalized rehabilitation plan focused on basic components of exercise and overall well-being, with limited specification of progression and intensity parameters.

**Figure 4:**
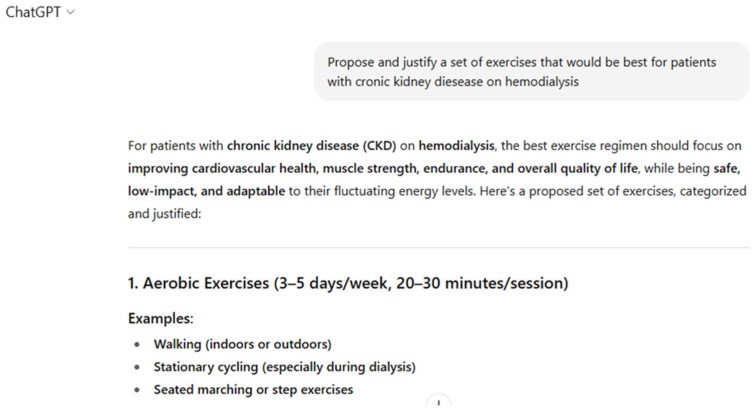
Example of an exercise program generated by ChatGPT in response to an English-language prompt. The output demonstrates improved clinical structuring, including defined training frequency, inclusion of balance exercises, and elements of intradialytic rehabilitation, with greater alignment with clinical recommendations.

**Figure 5:**
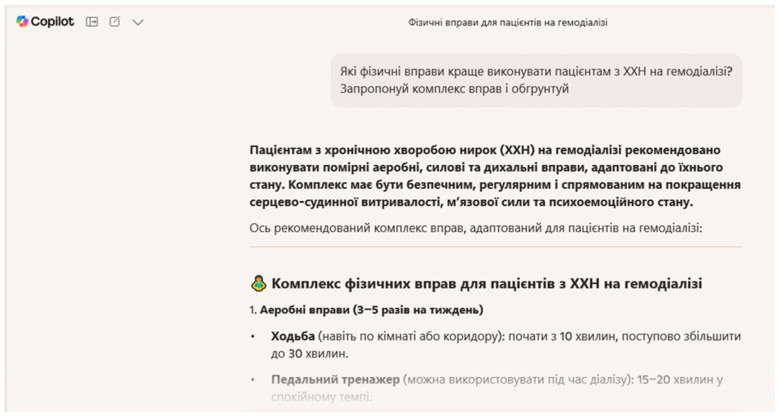
Example of an exercise program generated by Copilot in response to a Ukrainian-language prompt. The output includes core rehabilitation components and references to external sources, reflecting partial integration of domain-specific knowledge, although with limited clinical detailing compared to the English-language version.

**Figure 6:**
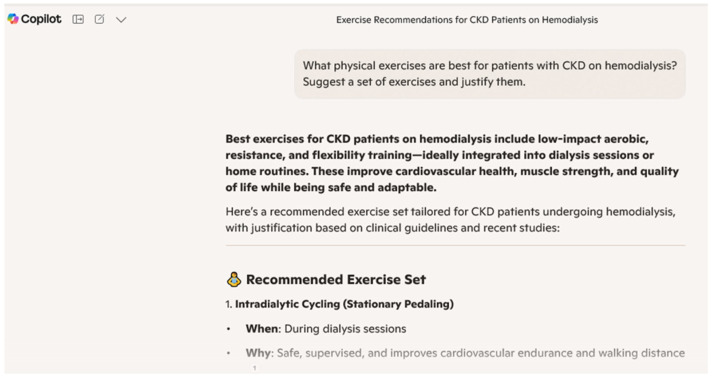
Example of an exercise program generated by Copilot in response to an English-language prompt. This output demonstrates the highest level of clinical precision among evaluated systems, including structured intradialytic exercise protocols, use of perceived exertion scales, and alignment with evidence-based rehabilitation principles.

Representative examples of AI-generated outputs are presented in Figure 1, Figure 2, Figure 3, Figure 4, Figure 5 and Figure 6.

### 3.1. General Characteristics of AI-Generated Programs

All AI systems produced exercise programs that included the core components of renal rehabilitation, such as aerobic activity, resistance training, flexibility exercises, and relaxation techniques. This demonstrates that large language models are capable of reproducing the general structure of rehabilitation protocols for CKD patients.

However, important differences emerged in the level of clinical detail and alignment with evidence-based recommendations. English-language outputs consistently demonstrated:

- explicit specification of training frequency, duration, and intensity;
- incorporation of validated monitoring tools (e.g., Borg Rating of Perceived Exertion scale);
- differentiation between exercise modalities (intradialytic vs. non-dialysis);
- detailed safety considerations, including vascular access protection and symptom monitoring.

In contrast, Ukrainian-language outputs were characterized by:

- more generalized and descriptive recommendations;
- absence or limited use of objective intensity metrics;
- reduced emphasis on structured progression;
- greater focus on overall well-being, fatigue reduction, and psychological support.

These findings suggest that while both language versions produced safe recommendations, their clinical applicability and alignment with guideline-based rehabilitation principles differed substantially.

### 3.2. Gemini Outputs

When prompted in Ukrainian, Gemini generated a program consisting of three main components: (1) aerobic activities such as walking, cycling, or swimming for 20–30 min, three to five times per week; (2) resistance exercises including chair rises, arm curls with light weights or water bottles, leg extensions, and elastic band training, recommended two to three times weekly; (3) daily stretching of the neck, shoulders, back, and lower limbs.

An example of the Ukrainian-language Gemini output is presented in Figure 1.

Although the Ukrainian-language program generated by Gemini demonstrated internal consistency and adhered to basic safety principles, it lacked several elements essential for structured renal rehabilitation. In particular, the output did not include detailed progression strategies, objective intensity markers, or explicit clinical monitoring parameters. As a result, the recommendations, while easy to understand and implement, were oriented primarily toward general physical activity rather than toward a structured, guideline-aligned rehabilitation protocol tailored to the needs of hemodialysis patients.

In contrast, the English-language output generated by Gemini demonstrated a substantially higher level of clinical detailing. The program included clearly defined progression steps, such as increasing aerobic activity from 10–15 min to 30–45 min over time, and specified moderate exercise intensity using the Borg Rating of Perceived Exertion scale (RPE 11–13). Resistance training was also presented in a more structured format, with explicit dosing parameters, including the number of sets and repetitions. These features reflect closer alignment with evidence-based rehabilitation frameworks and provide a more clinically actionable exercise prescription.

The English-language Gemini output is presented in Figure 2.

The English-language Gemini output included several clearly defined components that reflected closer adherence to evidence-based rehabilitation principles. These comprised:

- Aerobic training: low-impact walking (treadmill or outdoors), stationary or recumbent cycling, and water aerobics on non-dialysis days, beginning with 10–15 min and progressing toward 30–45 min at moderate intensity (RPE 11–13).
- Resistance training: light to moderate loads targeting major muscle groups (legs, arms, chest, back, shoulders), with explicit dosing parameters (1–2 sets of 8–12 repetitions) and at least one rest day between sessions.
- Flexibility training: daily static and dynamic stretches for the neck, shoulders, back, hips, and hamstrings to maintain joint mobility and reduce stiffness, tailored to the needs of patients undergoing hemodialysis.

Despite similarities in the types of exercises recommended across languages, the two versions differed substantially in their level of clinical refinement. Both programs emphasized several fundamental principles—beginning with low intensity and gradually increasing workload, the necessity of medical consultation, the importance of individualization, and the need to avoid excessive strain on the arm with an arteriovenous fistula (AVF) or catheter. These shared elements indicate that Gemini consistently incorporates core safety considerations regardless of language.

However, the English-language program was considerably more comprehensive and clinically detailed. In contrast, the Ukrainian-language version provided a more direct and simplified set of exercises, facilitating rapid orientation but offering less guidance on progression and monitoring. The English-language output also included an expanded section on “Important considerations,” addressing symptom monitoring (e.g., dyspnea, pain), medication effects, fluid and electrolyte balance, and motivational strategies. The Ukrainian-language version, by comparison, focused primarily on basic recommendations such as hydration and avoiding strenuous activity immediately after dialysis.

Taken together, both the Ukrainian- and English-language exercise programs generated by Gemini can be considered safe and appropriate for patients on hemodialysis, as they adhere to the core principles of low intensity, gradual progression, exercise variety, and medical supervision. The Ukrainian-language program is best suited as a simple introductory guide, offering clear and accessible examples of exercises. In contrast, the English-language program represents a more comprehensive, academically grounded, and clinically oriented protocol with greater applicability in structured rehabilitation settings.

### 3.3. ChatGPT Outputs

ChatGPT outputs demonstrated a similar pattern of language-dependent variation. The Ukrainian-language program consisted of five components: (1) breathing exercises (daily, 5–10 min) aimed at improving blood oxygenation and reducing stress; (2) warm-up activities (slow marching in place, circular movements of the arms, shoulders, and ankles) for 5–7 min; (3) aerobic training (walking in place or on a treadmill, stationary cycling, light stepping) for 10–15 min to enhance cardiovascular function; (4) strength exercises (chair squats, seated leg extensions and flexions, arm raises with light weights, forearm plank) for 10–15 min to prevent muscle atrophy; (5) stretching and relaxation (calf stretches, back stretches, relaxation techniques) for 5–10 min to improve flexibility, reduce the risk of muscle spasms, and support psychological well-being.

The structure was clear and clinically reasonable, with emphasis on improving cardiovascular function, preventing muscle atrophy, and enhancing psychological well-being.

An example of the Ukrainian-language ChatGPT output is presented in Figure 3.

However, the Ukrainian-language version lacked detailed progression strategies and did not include objective measures of exercise intensity. The recommendations were primarily descriptive and focused on general rehabilitation goals rather than on structured, measurable training parameters. As a result, while the program was safe and clinically reasonable, its applicability for individualized rehabilitation planning was limited.

When the same query was formulated in English, ChatGPT generated a more clinically structured program that also included aerobic and resistance exercises (Figure 4). In this version, flexibility and stretching were presented either as a distinct daily component or as part of post-exercise routines, with explicit inclusion of yoga-inspired movements. Breathing exercises were not listed as a separate category but were integrated into relaxation or yoga blocks, reflecting a more consolidated approach to mind–body elements.

In addition, the English-language program contained a dedicated section on intradialytic exercises—such as mini-cycle pedaling, seated leg lifts, and arm exercises with resistance bands—which was entirely absent from the Ukrainian-language version. This distinction is clinically relevant, as intradialytic training is a recognized component of modern renal rehabilitation and is supported by international guidelines.

The English-language ChatGPT output is presented in Figure 4.

Importantly, this version incorporated intradialytic exercise modalities, such as mini-cycle pedaling and seated resistance exercises, and included recommendations for intensity monitoring and progression.

### 3.4. Copilot Outputs

Copilot outputs showed the highest level of clinical precision among the evaluated systems. The Ukrainian-language version included (1) aerobic training three to five times per week (walking, pedal ergometer, recumbent cycling, or bed-based leg movements); (2) resistance training two to three times weekly (toe raises, chair squats, arm curls with light weights); (3) stretching and flexibility exercises (yoga, Pilates, hamstring and calf stretches); (4) breathing/relaxation techniques. The Ukrainian version highlighted psychological well-being and adaptation to patient condition but offered limited clinical detail.

In addition, Copilot provided references to relevant literature:

1. https://nephrocenter.com/novyny/fizychna-aktyvnist-na-dializi/ (accessed on 2 April 2026)
2. https://neonatology.bsmu.edu.ua/article/view/326117 [28] (accessed on 2 April 2026)
3. https://ua.xjcistanche.com/news/management-of-exercise-rehabilitation-in-patie-62436106.html (accessed on 2 April 2026)

The Ukrainian-language Copilot output is presented in Figure 5.

A notable feature of this output was the inclusion of external references, including scientific publications and educational materials, suggesting partial integration of domain-specific knowledge even in non-English contexts.

Nevertheless, the Ukrainian-language output remained less structured in terms of progression and clinical monitoring compared with the English-language version. The English-language Copilot output is presented in Figure 6.

It included structured intradialytic exercise protocols, precise workload assessment using the Borg scale, incorporation of balance training, and references to clinical guidelines.

### 3.5. Cross-System Comparative Analysis

A comparative analysis across all three AI systems revealed consistent and reproducible patterns in the influence of query language on the structure and clinical depth of the generated rehabilitation programs. Across platforms, English-language prompts resulted in outputs that were:

- more detailed and clinically explicit,
- better structured and more internally coherent,
- more closely aligned with international rehabilitation guidelines,
- more focused on measurable parameters, progression strategies, and monitoring tools.

In contrast, Ukrainian-language prompts produced outputs that were:

- simpler and more descriptive,
- less structured in terms of progression and dosing,
- less clinically detailed,
- more oriented toward general health promotion and psychological support.

These patterns were consistent across all three evaluated systems, although each model demonstrated characteristic strengths:

- Copilot generated the highest level of clinical precision, including structured intradialytic protocols, explicit workload assessment, and integration of guideline-based monitoring strategies.
- ChatGPT provided the most balanced and consistently structured outputs, with clear organization and comprehensive coverage of core rehabilitation components.
- Gemini emphasized accessibility, safety, and patient motivation, producing outputs that were easy to understand but less detailed in clinical parameters.

Together, these findings indicate that the observed language-dependent differences are not model-specific but reflect broader patterns in multilingual performance across contemporary large language models.

### 3.6. Integrated Rehabilitation Protocol

The integrated rehabilitation protocol presented below is not a direct study outcome but an author-derived synthesis based on comparative analysis of AI outputs. It is intended as an illustrative framework rather than a validated clinical intervention.

Based on the cross-system comparative analysis, a comprehensive rehabilitation protocol was developed by integrating the most clinically relevant elements from each AI system. This synthesis allowed the construction of a structured, evidence-informed program that reflects the strengths of all three models while addressing the limitations observed in individual outputs.

The integrated protocol included:

- aerobic, resistance, flexibility, balance, and intradialytic components,
- clearly defined training parameters (frequency, duration, intensity, progression),
- safety guidelines and monitoring strategies, including vascular access protection and symptom-based adjustments,
- principles of gradual progression, individualized dosing, and adaptation to dialysis schedules,
- emphasis on adherence and patient-centered care, including motivational strategies and fatigue management.

Recommended Exercise Set

- Intradialytic Cycling (Stationary Pedaling): during dialysis sessions to improve lower-limb endurance and reduce intradialytic discomfort.
- Resistance Band Training: during or outside dialysis (seated leg extensions, biceps curls, shoulder presses) to maintain muscle mass and functional strength.
- Walking: on non-dialysis days to support cardiovascular conditioning and daily activity levels.
- Stretching and Flexibility Exercises: daily or before/after other exercises (neck rolls, shoulder circles, hamstring stretches) to maintain joint mobility and reduce stiffness.
- Balance Training: 2–3 times per week (heel-to-toe walking, single-leg stands) to reduce fall risk and improve neuromuscular control.

Notably, Copilot also provided references to English-language literature, including clinical guidelines and educational resources:

1. https://academic.oup.com/ckj/article/17/7/sfae165/7690763 (accessed on 2 April 2026)
2. https://link.springer.com/article/10.1007/s40620-024-02049-9 (accessed on 2 April 2026)
3. https://www.kidney.org/sites/default/files/staying_fit_with_kidney_disease_1.pdf (accessed on 2 April 2026)
4. https://www.era-online.org/wp-content/uploads/2022/11/1.Physical-exercise-in-CKD-patients.pdf (accessed on 2 April 2026)

These sources further support the clinical relevance of the integrated protocol.

Overall, although both Ukrainian- and English-language exercise programs generated by the AI systems were safe, effective, and adapted to the needs of patients on hemodialysis, the Ukrainian-language outputs were more oriented toward general well-being and included simpler exercises and breathing techniques. In contrast, the English-language outputs were structured according to clinical protocols, with explicit workload assessment, emphasis on functional mobility, and strategies for fall prevention.

Based on this synthesis, a detailed comprehensive rehabilitation protocol was developed and is presented below in Table 1 and Table 2.

Safety & Monitoring Guidelines

- Avoid pressure on the vascular access site (fistula/graft/catheter)
- Use Borg RPE scale to guide intensity (target 11–13 for moderate effort)
- Warm-up and cool-down are essential (5–10 min each)
- Monitor symptoms: stop exercise if dizziness, chest pain, or shortness of breath occurs
- Coordinate with healthcare team: nephrologist, dialysis nurse, physiotherapist
- Hydration and medications should be managed per clinical guidance
- Progress gradually: increase duration, resistance, or complexity over time
- Encourage enjoyment and autonomy to improve adherence and mental well-being

## 4. Discussion

The present study demonstrates that the language of interaction is a clinically relevant determinant influencing the structure, completeness, and potential applicability of AI-generated rehabilitation recommendations for patients with chronic kidney disease (CKD). Across all evaluated platforms—Gemini, ChatGPT, and Copilot—English-language prompts consistently produced more detailed, structured, and guideline-aligned outputs, whereas Ukrainian-language prompts generated simplified, less clinically explicit, and more wellness-oriented recommendations. These findings indicate that linguistic factors may meaningfully shape the quality of AI-assisted clinical content.

The observed patterns align with emerging evidence on linguistic asymmetry in large language models (LLMs), which are predominantly trained on English-language corpora and therefore demonstrate higher performance in English compared with other languages [3,4,5,19,20,21]. Prior studies have shown that such asymmetry affects not only linguistic fluency but also clinical reasoning, completeness of recommendations, and adherence to evidence-based standards [20,21,23,24]. In healthcare applications, these differences may directly influence patient safety, the reliability of AI-assisted decision support, and the consistency of clinical guidance delivered across linguistic contexts.

From a nephrology perspective, these findings are particularly important. Renal rehabilitation requires individualized exercise prescription, structured progression, and strict safety considerations, including protection of vascular access and adaptation to dialysis schedules [10,11,12,13]. Contemporary evidence-based approaches emphasize multimodal exercise interventions and structured monitoring using validated tools such as the Borg scale [12,13,16,17]. Systematic reviews confirm that exercise interventions improve functional capacity and clinical outcomes in CKD patients [12,15]. Therefore, insufficient detail or incomplete safety framing in AI-generated recommendations may limit their clinical usefulness or, in some cases, introduce risk if used without expert oversight.

The results of this study are consistent with previous work by Bezruk et al. (2025), which demonstrated that AI-assisted development of rehabilitation programs in nephrology can achieve clinically meaningful structure and safety when aligned with evidence-based medicine principles [28]. At the same time, recent nephrology-focused AI research highlights both the potential and the limitations of LLMs in CKD management [7,22]. While these systems can support clinical reasoning and generate structured recommendations, their outputs remain sensitive to prompt formulation, training data distribution, and linguistic context. The present findings reinforce the need for careful evaluation of AI-generated content, particularly in languages with limited representation in training corpora.

Importantly, the differences identified in this study do not imply that AI-generated outputs in non-English languages are inherently unsafe. Rather, they reflect structural limitations of current AI systems and underscore the necessity of professional oversight. AI should therefore be considered an assistive clinical tool rather than a substitute for expert judgment [5,10,11]. Clinicians must remain aware of the variability in AI performance across languages and incorporate AI-generated recommendations into care pathways only after appropriate expert validation.

The findings also have broader implications for digital health equity. If AI systems systematically produce less detailed or less clinically robust outputs in non-English contexts, this may exacerbate disparities in access to high-quality healthcare information [21,23]. Recent studies emphasize that LLMs may reinforce existing inequities if linguistic biases are not addressed [24]. In regions where English is not the primary language of clinical communication, such disparities may limit the safe and effective integration of AI into routine nephrology practice.

These findings are consistent with recent multilingual LLM research demonstrating that model performance systematically declines in languages with limited representation in training corpora. Large comparative analyses show persistent structural inequalities across languages [29,30]. Additional work highlights tokenizer-level inefficiencies that disproportionately affect morphologically rich languages, including Ukrainian [31]. Importantly, clinical evaluations confirm that medical reasoning and safety framing degrade when prompts are issued in non-English languages, even when the underlying model remains the same [32]. Broader assessments of multilingual model behavior further emphasize the need for holistic evaluation frameworks to ensure equitable performance across linguistic contexts [33].

Additional nephrology literature supports the importance of structured and evidence-based rehabilitation strategies, highlighting the need for broader implementation and standardization in clinical practice [8,18]. The integration of outputs from multiple AI systems, as demonstrated in this study, represents a promising approach that allows the combination of complementary strengths to construct comprehensive rehabilitation protocols aligned with modern nephrology practice [8,16,17]. This integrative strategy may help mitigate individual model limitations and enhance the clinical utility of AI-generated recommendations.

These observations are consistent with current international policy frameworks addressing safe and equitable AI deployment in healthcare. The World Health Organization guidance and the European Union Artificial Intelligence Act emphasize transparency, safety, inclusivity, and the need to mitigate algorithmic bias [19,25]. The present findings reinforce the relevance of these principles in the context of multilingual clinical environments.

This study also highlights broader implications for policy and research. The development of multilingual AI systems and standardized evaluation frameworks is essential to ensure equitable clinical performance across healthcare systems [19,24]. Future research should prioritize multilingual benchmarking, expert-based evaluation frameworks, and prospective clinical trials assessing the real-world impact of AI-assisted rehabilitation strategies in CKD patients. Beyond immediate clinical application, the findings underscore the urgent need for the development of multilingual AI corpora and validated prompt libraries to reduce inequities in access to AI-assisted rehabilitation [19,24].

A pragmatic pathway to improving the effectiveness of AI systems in clinical practice is the use of English-language interaction design, particularly in settings where English-language outputs demonstrate higher clinical completeness [19,25]. This approach is especially relevant in the context of Ukraine’s prospective alignment with the European Artificial Intelligence Act (Regulation [EU] 2024/1689), which establishes harmonized requirements for safety, transparency, and reliability of AI systems in healthcare [19,25]. At the same time, future research should prioritize the clinical validation of AI-generated rehabilitation programs for patients with CKD from an evidence-based medicine perspective. Equally important is the development of multilingual AI frameworks and standardized prompt strategies aimed at reducing linguistic bias and ensuring equitable access to high-quality, AI-assisted rehabilitation. Such efforts are essential for transforming AI from a supportive tool into a reliable, safe, and globally accessible component of nephrology practice.

### Limitations

This study has several limitations. First, the analysis was qualitative and did not include quantitative scoring or statistical validation. Second, only a single standardized prompt was used in each language, which limits generalizability given the sensitivity of AI systems to prompt formulation. Third, although translation fidelity was ensured through forward and back-translation, semantic nuances may still have influenced the outputs. Fourth, evaluators were blinded to the AI system but not to the language of the output, which may introduce subjective bias. Fifth, no patient-level validation was performed, and the findings reflect structural rather than clinical effectiveness. Sixth, AI systems evolve rapidly, and the outputs represent a specific time point. Seventh, the study included only three AI systems and two languages; broader multilingual benchmarking is needed. Finally, tokenizer-level and model-architecture-level sources of linguistic bias were not assessed.

Subtle lexical differences between the Ukrainian and English prompts may have influenced model behavior, although both were validated through forward and back-translation. This protocol is not a validated clinical intervention and requires prospective evaluation before clinical implementation.

The integrated protocol requires prospective clinical validation before implementation and should not be interpreted as a guideline-level recommendation.

## 5. Conclusions

This study demonstrates that the language of the user query substantially influences the structure, clinical depth, and guideline alignment of AI-generated rehabilitation programs for patients with chronic kidney disease undergoing hemodialysis. English-language prompts consistently produced more detailed, clinically structured, and guideline-aligned outputs, whereas Ukrainian-language prompts generated simpler and more wellness-oriented recommendations. These findings highlight the need for improved multilingual training of large language models to ensure equitable access to AI-assisted healthcare across linguistic contexts. Further development of multilingual AI systems, together with standardized prompt frameworks and prospective clinical validation, will be essential to support safe and effective integration of generative AI into nephrology rehabilitation practice.

## Figures and Tables

**Table 1:** Exercise Components.

Type	Frequency	Examples	Clinical Rationale
Aerobic Training	3–5 times/week (non-dialysis or intradialytic)	Walking, stationary cycling, seated marching	Enhances cardiovascular function, blood pressure, lipid profile, and energy levels
Resistance Training	2–3 times/week (non-consecutive days)	Resistance bands, light dumbbells, chair squats, wall push-ups	Builds muscle mass, improves metabolism, supports bone density
Flexibility & Stretching	Daily or post-exercise	Hamstring stretch, shoulder rolls, yoga-inspired poses	Maintains joint mobility, reduces stiffness, supports posture and relaxation
Balance Training	2–3 times/week	Heel-to-toe walk, single-leg stands, tai chi drills	Reduces fall risk, improves coordination and neuromuscular control
Intradialytic Exercise	3 times/week during dialysis	Mini-cycle pedaling, seated leg lifts, arm curls with bands	Increases adherence, improves dialysis clearance, reduces sedentary time

**Table 2:** Sample Weekly Routine (Assuming Dialysis on Mon-Wed-Fri).

Day	Focus	Activities
Monday	Intradialytic Aerobic + Resistance	Pedal during dialysis, seated arm curls, avoid fistula arm
Tuesday	Walking + Stretching + Balance	20-min walk, full-body stretches, balance drills
Wednesday	Intradialytic Resistance + Flexibility	Resistance band exercises, gentle yoga post-dialysis
Thursday	Restorative + Flexibility	Light yoga or tai chi, deep breathing, stretching
Friday	Intradialytic Aerobic + Light Resistance	Seated cycling, leg extensions, cool-down stretches
Saturday	Outdoor Activity + Strength + Balance	Brisk walk, chair squats, wall push-ups, balance work
Sunday	Active Rest + Flexibility	Leisure walk, gardening, meditation, stretching

## Data Availability

The data presented in this study are available on request from the corresponding author.

## Supplementary Materials

The following supporting information can be downloaded at: https://www.mdpi.com/article/10.3390/jcm15114218/s1, Table S1: AI System Specifications; Table S2: Evaluation Rubric; Figure S1: Methodological Flow Diagram (text version); File S1: Full Prompts.

## Abbreviations

AVF	Arteriovenous Fistula
AI	Artificial Intelligence
CKD	Chronic Kidney Disease
KDIGO	Kidney Disease: Improving Global Outcomes
ERA	European Renal Association
ISN	International Society of Nephrology
RPE	Rating of Perceived Exertion
LLM	Large Language Model
