# Supplementary material for: Impact of Query Language on the Structure and Guideline Alignment of AI-Generated Rehabilitation Programs in Chronic Kidney Disease
Source: J Clin Med. 2026 May 29;15(11):4218. doi: 10.3390/jcm15114218 (PMC13257556; doi:10.3390/jcm15114218)
Supplement: Supplementary file 1 [file jcm-15-04218-s001.zip › jcm-4320355-supplementary.pdf]

Supplementary Materials for: "Impact of Query Language on the Structure and Guideline Alignment of AI-Generated Rehabilitation Programs in Chronic Kidney Disease"

Supplementary file S1.

Supplementary Table S1. AI System Specifications

AI System	Model Version (at time of access)	Access Date & Time	Default Temperature	Generation Mode
**Gemini**	Gemini 1.0 Pro	12 Feb 2026, 14:30 CET	Default (0.7)	Standard text generation
**ChatGPT**	GPT-4.1	12 Feb 2026, 15:10 CET	Default (0.7)	Standard mode
**Copilot**	GPT-4-class model	12 Feb 2026, 16:00 CET	Default	Balanced mode

Supplementary Table S2. Evaluation Rubric

Domain	Definition	Indicators	Source
**Guideline alignment**	Consistency with KDIGO/ERA/ISN rehabilitation principles	Evidence-based exercise types; dialysis-specific adaptations	KDIGO, ERA, ISN
**Clinical detail**	Specificity of prescription parameters	Frequency, duration, intensity, progression, monitoring tools	[7–9,22–24]
**Safety considerations**	Protection of vascular access and symptom monitoring	AVF protection, contraindications, red-flag symptoms	KDIGO
**Adaptability**	Ability to tailor program to dialysis schedule and patient needs	Intradialytic options, home-based modifications	ERA
**Structural completeness**	Inclusion of core rehabilitation components	Aerobic, resistance, flexibility, balance, relaxation	ISN

Supplementary Figure S1. Methodological Flow Diagram (text version)

Flowchart: Study Workflow

1. **Scientometric Review**
    - Identification of evidence-based CKD rehabilitation principles
    - Databases: PubMed, EMBASE, Scopus, Web of Science, Cochrane CENTRAL

2. **AI Interaction Phase**
    - Three AI systems queried (Gemini, ChatGPT, Copilot)
    - Standardized prompts in Ukrainian and English
    - Identical interaction conditions
3. **Output Extraction**
    - Collection of all generated exercise programs
    - Removal of duplicate or incomplete outputs
4. **Expert Evaluation**
    - Three independent evaluators
    - Blinded to AI system identity
    - Structured rubric applied
5. **Comparative Analysis**
    - Cross-language comparison
    - Cross-system comparison
    - Thematic synthesis

Supplementary File S1. Full Prompts

Ukrainian prompt

«Запропонуйте безпечний комплекс фізичних вправ для пацієнтів із ХХН на гемодіалізі».

English prompt

"Please propose a safe and effective exercise program for patients with CKD on hemodialysis."
